# Association between thyroid hormones and diabetic kidney disease in Chinese adults

**DOI:** 10.1186/s12902-023-01299-1

**Published:** 2023-03-08

**Authors:** Meng-chao Liu, Jia-lin Li, Yue-fen Wang, Yuan Meng, Zhen Cai, Cun Shen, Meng-di Wang, Wen-jing Zhao, Wen-quan Niu

**Affiliations:** 1 Department of Nephropathy, Beijing Traditional Chinese Medicine Hospital, Capital Medical University, Beijing, China; 2 Institute of Clinical Medical Sciences, China-Japan Friendship Hospital, Beijing, China; 3 Beijing University of Chinese Medicine, Beijing, China

**Keywords:** Thyroid hormones, Diabetic kidney disease, Diabetes mellitus, Association, Nomogram

## Abstract

**Objective:**

We aimed to explore the association between thyroid hormones and different stages of diabetic kidney disease (DKD) in Chinese adults.

**Methods:**

This is a retrospective study involving 2,832 participants. DKD was diagnosed and classified according to the Kidney Disease: Improving Global Outcomes (KDIGO) categories. Effect sizes are expressed as odds ratio (OR) with 95% confidence interval (CI).

**Results:**

After propensity score matching (PSM) on age, gender, hypertension, hemoglobin A1c(HbA1c), total cholesterol (TC), serum triglyceride (TG) and duration of diabetes, per 0.2 pg/mL increment in serum free triiodothyronine (FT3) was significantly associated with 13%, 22% and 37% reduced risk of moderate-risk (OR, 95% CI, P: 0.87, 0.70–0.87, < 0.001), high-risk (0.78, 0.70–0.87, < 0.001) and very-high-risk (0.63, 0.55–0.72, < 0.001) DKD stages relative to the low-risk DKD stage, respectively. After PSM analyses, serum FT4 and TSH showed no statistical significance in risk estimates for all DKD stages. To facilitate clinical application, a nomogram prediction model was established for the moderate-risk, high-risk and very-high-risk DKD stages, with decent accuracy.

**Conclusion:**

Our results indicate that high concentrations of serum FT3 were associated with the significantly reduced risk of having moderate-risk to very-high-risk DKD stages.

## Introduction

Diabetic kidney disease (DKD) has become the leading cause of kidney failure around the world, accounting for approximately 50% of cases in developed countries [1, 2]. Recently, besides classic manifestations, some patients with DKD present with novel phenotypes, such as nonalbuminuric renal impairment and progressive renal decline, which necessitate an integrated consideration of proteinuria levels and glomerular filtration rate (GFR) in patients with DKD [3, 4]. In 2012, the Kidney Disease: Improving Global Outcomes (KDIGO) addressed a new classification of chronic kidney disease (CKD), viz. “risk categories” [5], a combination of GFR and albuminuria [6], that has been applied widely to classify DKD in routine clinical practice [7]. This classification was once again underscored by the KDIGO Diabetes Work Group in 2020 [8]. Despite the use of existing lifestyle and pharmacological therapies, patients with diabetes mellitus are still at high risk for developing DKD [5]. Therefore, it is of clinical importance to identify which biomarkers can accurately predict the progression of DKD.

As one of the most important endocrine hormones in the human body, thyroid hormones have become a hot point of DKD-related research in recent years. A large number of studies have confirmed that hypothyroidism or subclinical hypothyroidism in patients with DKD has significantly increased in recent years [9–11], and diabetic patients with hypothyroidism have a higher risk of DKD and DR [10]. In a study of 146 patients diagnosed with diabetic nephropathy (DN) by renal biopsy, patients with high thyroid-stimulating hormone (TSH) or low free triiodothyronine (FT3) were found to have more severe proteinuria, renal insufficiency and glomerulonpathy [12]. Additionally, in an attempt to distinguish the effect of thyroid hormones on other kidney diseases, Bando and colleagues conduced a meta-analysis and found that patients with advanced DKD had a significantly higher prevalence of non-autoimmune primary hypothyroidism than patients with other types of renal insufficiency [13]. However, there is no consensus on the relationship between each thyroid hormone and DKD progression, and the association between thyroid hormones and DKD using the KDIGO risk categories is rarely reported in the medical literature.

To fill this gap in knowledge, we conducted a retrospective study to explore the association between thyroid hormones and different DKD stages in Chinese adults.

## Methods

### Study participants

Participants aged 18 to 80 were chronologically and retrospectively recruited from Beijing Hospital of Traditional Chinese Medicine affiliated to Capital Medical University from January, 2011 to January 2021. Initially, a total of 16,589 patients with type 2 diabetes mellitus were enrolled in this study. After screening, 13,757 patients were excluded for the following reasons: (i) receiving renal replacement therapies; (ii) having a definite diagnosis of nondiabetic kidney disease; (iii) having diabetic acute complications, such as diabetic ketoacidosis or hyperosmolar hyperglycemic coma; (iv) having chronic diseases that may affect metabolic function, including hypothalamic disease, adrenal disease, any thyroid medication (levothyroxine or antithyroid medication), or a history of thyroid diseases prior to diabetes mellitus; (v) having severe respiratory, digestive, or hematological diseases, or current acute or severe infections, autoimmune diseases, or malignancies; (vi) lacking necessary information, such as serum creatinine and urinary albumin-to-creatinine ratio (UACR). Finally, total 2,832 eligible patients were enrolled in the present study, including 1,710 males and 1,122 females.

### Diagnosis of DKD

According to the 2012 KDIGO clinical practice guidelines, DKD was defined as urinary albumin/creatinine ratio (UACR) ≥ 30 mg/g, or an estimated glomerular filtration rate (eGFR) < 60 mL/min/1.73 m^2^ in the absence of signs or symptoms of other primary causes of kidney damage [6]. The eGFR was estimated based on the 2009 CKD Epidemiology Collaboration (CKD-EPI) equation [14].

### Stages of DKD

The DKD patients were divided into four stages according to the KDIGO risk categories, that is, low risk, moderate risk, high risk and very-high risk [5]. The KDIGO risk categories have two components: persistent albuminuria categories and GFR categories. The GFR categories include G1 (GFR in ml/min/1.73m^2^: ≥ 90), G2 (60–89), G3a (45–59), G3b (30–44), G4 (15–29) and G5 (≤ 15 or treatment by dialysis). The persistent albuminuria categories include A1 (ACR in mg/g: ≤30), A2 (30–299) and A3 (≥ 300). The G3a and G3b categories were combined into G3 due to limited sample sizes.

### Clinical and biochemical indices

Data analyzed in this study were applied and abstracted from the scientific research sharing platform (Yidu Cloud Research Collaboration Platform) of Beijing Hospital of Traditional Chinese Medicine affiliated to Capital Medical University, and all study participants were given standardized questionnaires for demographics and medical histories. Diabetes mellitus was defined as fasting plasma glucose ≥ 7.0 mmol/L, or 2-h plasma glucose ≥ 11.1mmol/l during an OGTT, or hemoglobin A1c (HbA1c) ≥ 6.5%, or random plasma sugar ≥ 11.1mmol/l, or the acts of taking hypoglycemic drugs or receiving parenteral insulin therapy [15]. Hypertension was defined as systolic blood pressure (SBP) ≥ 140 mmHg or diastolic blood pressure (DBP) ≥ 90 mmHg or receiving antihypertensive medication [16]. Body mass index (BMI) was calculated as weight in kilograms divided by height in meters squared. Laboratory tests were performed for serum samples obtained by venipuncture after fasting for 8 h in patients prior to clinical treatment. Concentrations of serum thyroid hormone were measured by chemiluminescence, including triiodothyronine (T3), thyroxine (T4), FT3, free thyroxine (FT4) and TSH. The corresponding reference ranges used for these hormones were 0.60–1.81 ng/ml, 4.50–10.90 ug/dL, 2.30–4.20 pg/mL and 0.89–1.76 ng/dL, and 0.51–6.27 uIU/mL. Creatinine concentrations were determined by the enzymatic method, and urine microalbumin was determined by the immunoturbidimetric method. Serum triglycerides (TG), total cholesterol (TC), high density lipoprotein cholesterol (HDL-C), and low density lipoprotein cholesterol (LDL-C) concentrations were measured with an automated biochemical analyzer. HbA1c was determined by high performance liquid chromatography. All tests were measured twice before reporting and performed by trained staff at the laboratory of the Beijing Hospital of Traditional Chinese Medicine, Capital Medical University.

### Statistical analysis

Statistical analysis was completed with STATA version 16 (StataCorp, College Station, TX, USA). Normally distributed continuous variables are expressed as mean (standard deviation), skewed continuous variables as median (interquartile range), and categorical variables as count (percent). Between-group differences were assessed with χ^2^ test for categorical variables and with Wilcoxon rank sum test for continuous variables. The association between thyroid hormones and DKD stages before and after adjustment for confounders was examined using logistic regression analysis, with effect sizes expressed as odds ratio (OR) and 95% confidence interval (95% CI). Potential bias in group-based equivalents was controlled using propensity score matching (PSM). Calibration was assessed with Akaike Information Criterion (AIC), Bayesian Information Criterion (BIC), and − 2 log likelihood ratio tests. Discrimination was judged by net reclassification improvement (NRI) and integrated differential improvement (IDI). The association between thyroid hormones, UACR, and eGFR was evaluated with Spearman correlation analysis. Nomogram was made using the “RMS” package in the R programming environment (version 3.5.2). P values of less than 0.05 were considered statistically significant. Study power was estimated by adopting the PS Power and Sample Size Calculations software (version 3.0).

## Results

### Characteristics of subjects

Table 1 shows the baseline characteristics of study participants. Patients in low-risk DKD stage were used as a reference group (controls), and they were younger than patients in moderate to very high risk stages. Sex differed significantly between patients in lower-risk and high-risk stages. Compared with controls, duration of diabetes and percentages of diabetic retinopathy and hypertension were higher in patients in high-risk stages. For thyroid hormones, FT3 concentrations decreased gradually with the increasing severity of DKD. No significance was noted for T3, T4 and TSH between patients in moderate-risk stage and controls.

**Table 1:** Baseline characteristics of patients stratified by KDIGO categories

Characteristics	Patients with diabetes mellitus				
	Total (N = 2832)	Low risk (N = 736)	Moderate risk (N = 501)	High risk (N = 578)	Very high risk (N = 1017)
Age (years)	61 (53–69)	58 (51–66)	62 (55–70)^**^	61 (53–69)^**^	62 (54–70)^**^
Female, n (%)	1122 (39.62)	340 (46.20)	208 (41.50)	200 (34.60)^**^	374 (36.80)^**^
Duration of diabetes (yrs)	10 (6–20)	7 (3–12)	10 (5–16)^**^	10 (5–18)^**^	14.5 (9–20)^**^
Diabetic retinopathy, n (%)	990 (34.96)	122 (16.60)	125 (25.00)^**^	217 (37.50)^**^	526 (51.70)^**^
Hypertension, n (%)	2045 (72.20)	256 (64.60)	350 (71.30)^*^	483 (83.60)^**^	956 (94.00)^**^
SBP (mmHg)	140 (129–155)	130 (120–140)	135 (125–148)^**^	140 (130–155)^**^	150 (139–165)^**^
DBP (mmHg)	80 (74–90)	80 (71–85)	80 (72–88)	80 (75–90)^**^	80 (75–90)^**^
BMI (kg/m^2^)	26.00 (23.40–28.40)	26.20 (24.20–29.20)	26.05 (23.90–29.00)	25.30 (22.75–28.20)	25.70 (23.10–28.10)
HbA1c (%)	7.1 (6.2–8.6), 0.24	7.5 (6.5–9.1)	7.6 (6.6–9.3)	7.3 (6.4-9.0)	6.6 (5.9–7.7)^**^
TG (mmol/L)	1.67 (1.19–2.42), 0.86	1.54 (1.11–2.18)	1.56 (1.14–2.28)	1.77 (1.25–2.56)^**^	1.72 (1.24–2.52)^**^
TC (mmol/L)	4.77 (3.97–5.73), 0.36	4.64 (3.93–5.38)	4.57 (3.77–5.34)	4.90 (4.10–6.15)^**^	4.88 (3.99–6.12)^**^
LDL-C (mmol/L)	2.75 (2.15–3.46), 0.42	2.66 (2.12–3.26)	2.61 (2.03–3.29)	2.87 (2.22–3.71)^**^	2.85 (2.19–3.69)^**^
HDL-C (mmol/L)	1.17 (0.99–1.38), 0.31	1.19 (1.01–1.37)	1.12 (0.97–1.32)^**^	1.19 (0.99–1.42)	1.16 (0.97–1.39)
eGFR (ml/min/1.73 m²)	73.6 (36.9–99.4), 0.57	100.2 (91.4-108.9)	91.8 (73.6-103.6)^**^	82.6 (64.4–100.0)^**^	25.1 (12.2–39.7)^**^
UACR (mg/g)	309.06 (22.96-2182.22), 3.40	9.24 (4.96–15.91)	75.83 (39.50-148.20)^**^	957.54 (399.50-2444.80)^**^	2251.14 (869.76-4418.82)^**^
sALB (mg/L)	37.9 (33.3–41.8), 0.21	41.2 (38.2–44.2)	40.2 (37.5–43.5)^**^	36.0 (30.4–40.4)^**^	34.3 (28.8–38.5)^**^
T3 (ng/mL)	0.90 (0.77–1.04), 0.34	0.98 (0.85–1.09)	0.94 (0.81–1.08)	0.91 (0.79–1.07)^**^	0.83 (0.70–0.96)^**^
T4 (µg/dL)	8.00 (6.70–9.20), 0.25	8.27 (7.10–9.20)	8.20 (7.00-9.30)	7.79 (6.60–8.98)^**^	7.90 (6.50–9.20)^*^
FT3 (pg/mL)	2.73 (2.43–3.02), 0.18	2.95 (2.71–3.20)	2.86 (2.62–3.07)^**^	2.76 (2.50–3.05)^**^	2.45 (2.19–2.70)^**^
FT4 (ng/dL)	1.15 (1.02–1.28), 0.20	1.18 (1.06–1.31)	1.22 (1.07–1.34)^*^	1.17 (1.03–1.30)	1.10 (0.98–1.23)^**^
TSH (µIU/mL)	1.91 (1.25–3.32), 2.54	1.70 (1.10–2.65)	1.61 (1.09–2.53)	2.01 (1.24–3.24)^**^	2.24 (1.46–4.11)^**^

Abbreviations:

yrs, years; SBP, systolic blood pressure; DBP, diastolic blood pressure; BMI, body mass index; HbA1c, hemoglobin A1c; TG, serum triglyceride; TC, total cholesterol; LDL-C, low-density lipoprotein cholesterol; HDL-C, high-density lipoprotein cholesterol; eGFR, estimated glomerular filtration rate; UACR, urinary albumin-to-creatinine ratio; sALB, serum albumin; T3, triiodothyronine; T4, total thyroxine; FT3, free triiodothyronine; FT4, free thyroxine; TSH, thyroid-stimulating hormone. Continuous variables are expressed as median (interquartile range), and categorical variables as count (percent). Between-group comparison was done using Wilcoxon rank sum test or ^2^ test, where appropriate. *P < 0.05; **P < 0.01. In total patients, coefficient of variation (CV) was provided after median (interquartile range)

### Correlation analysis

Since free thyroid hormones are the physiologically active forms of thyroid hormones, only FT3, FT4, and TSH were examined in subsequent analyses. Table 2 shows the correlations of serum FT3, FT4 and TSH across persistent albuminuria categories and GFR categories.

**Table 2:** Correlations between serum thyroid Hormones and persistent albuminuria categories and GFR categories

	FT3	FT4	TSH
Persistent albuminuria categories	-0.290	-0.112	0.210
	P < 0.001	P < 0.001	P < 0.001
GFR categories	-0.490	-0.178	0.197
	P < 0.001	P < 0.001	P < 0.001

Abbreviations: FT3, free triiodothyronine; FT4, free thyroxine; TSH, thyroid-stimulating hormone. The correlation between thyroid hormones, UACR, and eGFR was evaluated using Spearman correlation analysis

### Thyroid hormones and DKD stages

Table 3 shows the association between thyroid hormones and DKD stages before and after PSM analyses by balancing age, gender, hypertension, HbA1c, TC, TG and duration of diabetes. Multiple comparisons were adjusted by Bonferroni correction method, with P values less than 0.05/9 indicating statistical significance. Before PSM analyses, per 0.2 pg/mL increment in serum FT3 was significantly associated with 13%, 22% and 37% reduced risk of moderate-risk (OR, 95% CI, P: 0.87, 0.70–0.87, < 0.001), high-risk (0.78, 0.70–0.87, < 0.001) and very-high-risk (0.63, 0.55–0.72, < 0.001) DKD stages relative to the low-risk DKD stage, respectively. After PSM analyses, serum FT4 and TSH showed no statistical significance in risk estimates for all DKD stages.

**Table 3:** Effect-size estimates of serum thyroid hormones with various stages of DKD

Significant risk factors	Low risk	Moderate risk	High risk	Very high risk
FT3 (+ 0.2 pg/mL)	Reference	0.87, 0.81 to 0.93, < 0.001	0.79, 0.74 to 0.85, < 0.001	0.56, 0.52 to 0.61, < 0.001
FT4 (+ 0.2 ng/dL)	Reference	1.20, 1.06 to 1.38, 0.005	1.04, 0.92 to 1.18, 0.491	0.82, 0.73 to 0.92, 0.001
TSH (+ 0.5 µIU/mL)	Reference	1.00, 0.99 to 1.00, 0.394	1.00, 0.99 to 1.00, 0.378	1.00, 1.00 to 1.01, 0.344
**After balancing age, gender, hypertension, HbA1c, TC, TG and duration of diabetes**				
FT3 (+ 0.2 g/mL)	Reference	0.87, 0.70 to 0.87, 0.006	0.78, 0.70 to 0.87, < 0.001	0.63, 0.55 to 0.72, < 0.001
FT4 (+ 0.2 ng/dL)	Reference	1.24, 1.04 to 1.50, 0.015	1.09, 0.93 to 1.30, 0.277	0.93, 0.78 to 1.11, 0.418
TSH (+ 0.5 µIU/mL)	Reference	0.99, 0.97 to 1.02, 0.352	0.99, 0.96 to 1.01, 0.264	0.99, 0.98 to 1.01, 0.329

Abbreviations: FT3, free triiodothyronine; FT4, free thyroxine; TSH, thyroid-stimulating hormone. Data are expressed as odds ratio, 95% confidence interval, P value

The power to reject null hypotheses with OR equal to 1 was over 80% for above significant comparisons.

### Prediction accuracy assessment

Table 4 shows an evaluation on the prediction accuracy after adding FT3 to the basic model that included age, gender, hypertension, HbA1c, TC, TG and duration of diabetes. The predicted probabilities of FT3 additions reflected the actual observed risk. In terms of calibration, reduction in AIC and BIC statistics was greater than 10 after the addition of FT3 to the basic model, for each stage. Moreover, the likelihood ratio test showed that the difference was statistically significant with FT3 for all stages.

**Table 4:** Prediction accuracy for DKD gained by adding thyroid hormones to the basic model for different stages

Statistics		Moderate risk			High risk			Very high risk	
	Basic model		Basic model + FT3	Basic model		Basic model + FT3	Basic model + FT3		Basic model
**Calibration**									
AIC	768.23		672.5	762.08		658.71	834.96		612.96
BIC	803.02		710.54	797.75		697.86	874.19		656.11
LR test (χ2)		12.2			29.46			127.32	
LR test P value		0.0005			< 0.001			< 0.001	
**Discrimination**									
NRI (P value)		< 0.001			0.003			< 0.001	
IDI (P value)		< 0.001			< 0.001			< 0.001	

Abbreviations: AIC, Akaike information criterion; BIC, Bayesian information criterion; LR, likelihood ratio; NRI net reclassification improvement; IDI, integrated discrimination improvement; FT3, free triiodothyronine

### Nomogram prediction model

**Fig. 1:**
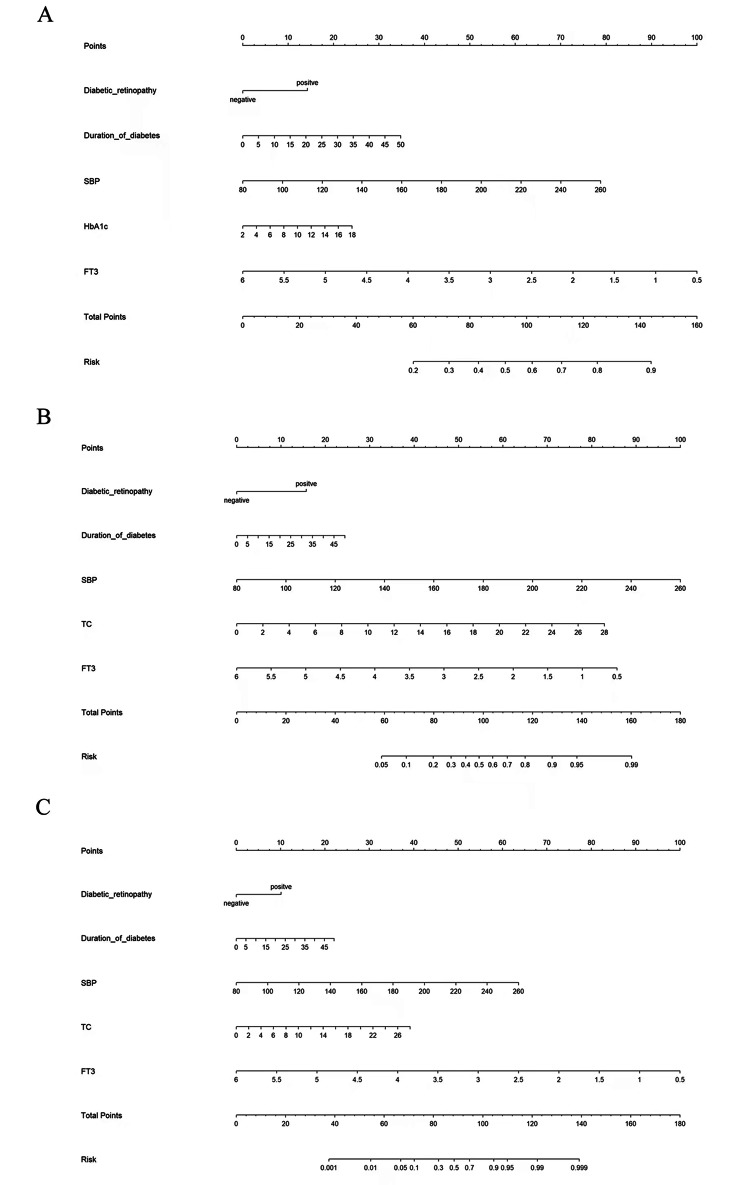
Nomogram prediction models for the moderate-risk (panel A), high-risk (panel B), and very-high-risk stages (panel C) Abbreviations: SBP, systolic blood pressure; HbA1c, hemoglobin A1c; FT3, free triiodothyronine; TC, total cholesterol

To facilitate clinical application, a nomogram prediction model was established for the moderate-risk, high-risk and very-high-risk DKD stages, as illustrated in Fig. 1. The predictive accuracy and discriminative capability of FT3 for each DKD stage were assessed by C-index (C-index: 0.673, P < 0.001 for moderate-risk stage; C-index: 0.810, P < 0.001 for high-risk stage; and C-index: 0.907, P < 0.001 for the very-high-risk stage), indicating significant improvement in model performance.

For example, on the basis of nomogram models, considering a patient with type 2 diabetes who has a 10-year history of diabetes but has not yet been evaluated for renal impairment, has no diabetic retinopathy, and has SBP at 140 mmHg, TC at 4.5 mmol/L, and FT3 at 4.2 pg/mL, the odds of having moderate-risk DKD was estimated to be 78%, high-risk DKD to be 84% and very-high-risk DKD to be 60%.

## Discussion

The aim of this retrospective study was to explore the association between thyroid hormones and different DKD stages in Chinese adults. Our key findings are that high concentrations of serum FT3 were associated with the significantly reduced risk of having moderate-risk to very-high-risk DKD stages, which supported the hypothesis that serum FT3 may serve as a promising biomarker to predict DKD progression.

Our findings of serum FT3 and DKD progression are biologically plausible. Thyroid hormones can affect renal development, glomerular and tubular function and renal hemodynamics, and activate the renin-angiotensin-aldosterone system [17, 18], and they may affect renal functions through cardiovascular and systemic hemodynamics in addition to acting directly on the kidney [19]. However, the kidney regulates thyroid hormone metabolism and elimination by promoting the removal of iodine through glomerular filtration [17], and elevated serum concentrations of inorganic iodide and thyroid iodine in patients with kidney disease can prolong the Wolff-Chaikoff effect and promote hypothyroidism [20]. Additionally, thyroxine binds tightly to protein and can be lost in urine of patients with kidney disease, which is commonly seen in patients with DKD. Furthermore, as effective regulators of glucose metabolism, thyroid hormones can also act in the development of diabetes mellitus, enhance the expressions of GK and Mafa in the pancreas, facilitate the rapid maturation and renewal of β-cells, and strengthen the expression and secretion of insulin in the pancreas [21, 22], which may affect the development of DKD. In particular, FT3 may be related to DKD through several mechanisms. Aggravated inflammation may lead to a decrease in FT3, and inflammatory cytokines such as tumor necrosis factor (TNF)-α and interleukin (IL)-1 can inhibit the expression of type 1 5’-deiodase and reduce the transformation of T4-to-T3 [29]. It is thus reasonable to infer that unfavorable changes in serum thyroid hormones, notably FT3, may worsen renal function, which then indicate the development and progression of DKD.

In recent years, some studies have explored the relationship between thyroid hormones and DKD, yet the results of these studies are not often reproducible. In a cross-sectional study involving 862 diabetic patients, FT3 in the normal range was negatively correlated with DKD in patients with type 2 diabetes mellitus and there was no correlation between FT4, TSH and DKD, consistent with the findings of the present study; however, the relationship between FT3 and different stages of DKD was not examined [23]. Another cross-sectional study involving 1,071 patients with type 2 diabetes mellitus revealed that after adjusting for covariates, serum FT3 and FT4 were negatively correlated with DKD and serum TSH was positively correlated with DKD [24]. In addition, the relationship between serum TSH and DKD also showed a positive correlation [25]. Other studies also have shown that low concentrations of normal FT3 were associated with a higher incidence of microalbuminuria [27]. In this present study, we focused on northern Chinese adults and employed the propensity score matching method to reduce possible selection bias. Our findings indicated that serum FT4 was negatively correlated with persistent albuminuria catagories and GFR categories and TSH was positively correlated with these categories as well, yet this result was not statistically significant after balancing confounders. These controversial findings may have thus resulted from the confounding factors of the study participants.

Moreover, we also found that FT3 decreased with the increase in DKD risk category, consistent with the results of several studies [23, 26]. We also constructed nomogram prediction models for DKD in different stages in an attempt to apply our findings to routine clinical practice and facilitate clinical decision making.

This study has some strengths. Following the recommendations of the 2020 KDIGO Diabetes Working Group, we first applied the KDIGO risk categories, combining eGFR and UACR to examine the association between thyroid hormone and DKD risk. Currently, few studies existed on the relationship between thyroid hormones and DKD even though thyroid hormone testing is commonly used and easy to obtain, and treatment methods for thyroid dysfunction are readily available. In addition, our study has a relatively large sample, and the collected data was relatively standardized, complete, and reliable.

However, our study has several limitations that need to be addressed. First, this is a retrospective analysis, and so we were only able to evaluate association, not causation. Further prospective and longitudinal studies are needed to determine the correlation between thyroid hormones and DKD more directly. Second, information on drug regimens, such as hypoglycemic drugs or antihypertensive drugs was not available for us, which left their potential contribution to the association between thyroid hormones and DKD an open question. Third, this study only involved Chinese adults, and so extrapolation to other ethnic groups or races may be limited. Fourth, study participants were patients from a single hospital. Although the study was strictly screened, there may still have been some unknown confounding factors that may have led to bias.

Despite these limitations, our findings indicate that high concentrations of serum FT3 were associated with the significantly reduced risk of having moderate-risk to very-high-risk DKD stages. We hope that this study can provide some clinical evidence for the association between thyroid hormones and DKD, and importantly lay a foundation for further research on the potential therapeutic effects of thyroid hormones on DKD.

## Data Availability

The datasets used and/or analysed during the current study are available from the corresponding author on reasonable request.

## References

[1] de Boer IH, Rue TC, Hall YN et al. Temporal trends in the prevalence of diabetic kidney disease in the United States. JAMA. 2011 Jun 22;305(24):2532-9. 10.1001/jama.2011.861. PMID: 21693741; PMCID: PMC3731378. 10.1001/jama.2011.861 PMC3731378 21693741

[2] Tuttle KR, Bakris GL, Bilous RW et al. Diabetic kidney disease: a report from an ADA Consensus Conference. Diabetes Care. 2014;37(10):2864–2883. doi:10.2337/dc14-1296 10.2337/dc14-1296 PMC4170131 25249672

[3] Kramer HJ, Nguyen QD, Curhan G et al. Renal insufficiency in the absence of albuminuria and retinopathy among adults with type 2 diabetes mellitus. JAMA. 2003 Jun 25;289(24):3273-7. 10.1001/jama.289.24.3273. PMID: 12824208. 10.1001/jama.289.24.3273 12824208

[4] Dai Q, Chen N, Zeng L, Lin XJ, Jiang FX, Zhuang XJ, Lu ZY. Clinical features of and risk factors for normoalbuminuric diabetic kidney disease in hospitalized patients with type 2 diabetes mellitus: a retrospective cross-sectional study. BMC Endocr Disord. 2021 May 22;21(1):104. 10.1186/s12902-021-00769-8. PMID: 34022855; PMCID: PMC8141213. 10.1186/s12902-021-00769-8 PMC8141213 34022855

[5] Goldenberg RM, Berall M, Chan CTM, et al. Managing the course of kidney disease in adults with type 2 diabetes: from the Old to the New. Can J Diabetes. 2018 Jun;42(3):325–34. 10.1016/j.jcjd.2017.06.008. Epub 2017 Aug 16. PMID: 28822777. 10.1016/j.jcjd.2017.06.008 28822777

[6] Kidney Disease (2013). Improving global outcomes (KDIGO) CKD Work Group KDIGO 2012 clinical practice guideline for the evaluation and management of chronic kidney disease. Kidney Int Suppl.

[7] Furuichi K, Shimizu M, Hara A et al. Diabetic Nephropathy: A Comparison of the Clinical and Pathological Features between the CKD Risk Classification and the Classification of Diabetic Nephropathy 2014 in Japan. Intern Med. 2018 Dec 1;57(23):3345–3350. 10.2169/internalmedicine.1132-18. Epub 2018 Aug 10. PMID: 30101924; PMCID: PMC6306527. 10.2169/internalmedicine.1132-18 PMC6306527 30101924

[8] Kidney Disease: Improving Global Outcomes (KDIGO) Diabetes Work Group. KDIGO 2020 Clinical Practice Guideline for Diabetes Management in Chronic Kidney Disease. Kidney Int. 2020 Oct;98(4S):S1-S115. 10.1016/j.kint.2020.06.019. PMID: 32998798. 10.1016/j.kint.2020.06.019 32998798

[9] Xie J, Wang X, Zhang Y et al. The longitudinal effect of subclinical hypothyroidism on urine microalbumin-to-urine creatinine ratio in patients with type 2 diabetes mellitus. BMC Endocr Disord. 2019 Aug 5;19(1):84. 10.1186/s12902-019-0405-0. PMID: 31382952; PMCID: PMC6683563. 10.1186/s12902-019-0405-0 PMC6683563 31382952

[10] Chen HS, Wu TE, Jap TS et al. Subclinical hypothyroidism is a risk factor for nephropathy and cardiovascular diseases in Type 2 diabetic patients. Diabet Med. 2007 Dec;24(12):1336-44. 10.1111/j.1464-5491.2007.02270.x. Epub 2007 Oct 17. Erratum in: Diabet Med. 2008 Feb;25(2):244. PMID: 17941864. 10.1111/j.1464-5491.2007.02270.x 17941864

[11] Furukawa S, Yamamoto S, Todo Y (2014). Association between subclinical hypothyroidism and diabetic nephropathy in patients with type 2 diabetes mellitus. Endocr J.

[12] Han Q, Zhang J, Wang Y et al. Thyroid hormones and diabetic nephropathy: An essential relationship to recognize. Nephrology (Carlton). 2019 Feb;24(2):160–169. 10.1111/nep.13388. PMID: 29660205. 10.1111/nep.13388 29660205

[13] Bando Y, Ushiogi Y, Okafuji K, et al. Non-autoimmune primary hypothyroidism in diabetic and non-diabetic chronic renal dysfunction. Exp Clin Endocrinol Diabetes. 2002 Nov;110(8):408–15. 10.1055/s-2002. 10.1055/s-2002-36427 12518252

[14] Palacio-Lacambra ME, Comas-Reixach I, Blanco-Grau A, et al. Comparison of the Cockcroft-Gault, MDRD and CKD-EPI equations for estimating ganciclovir clearance. Br J Clin Pharmacol. 2018 Sep;84(9):2120–8. 10.1111/bcp.13647. Epub 2018 Jul 8. PMID: 29791023; PMCID: PMC6089827. 10.1111/bcp.13647 PMC6089827 29791023

[15] American Diabetes Association. Diagnosis and classification of diabetes mellitus. Diabetes Care. 2013 Jan;36(1):67–74. 10.2337/dc13-S067. PMID: 23264425; PMCID: PMC3537273. 10.2337/dc13-S067 PMC3537273 23264425

[16] Liu LS, Writing Group of 2010 Chinese Guidelines for the Management of Hypertension. [2010 chinese guidelines for the management of hypertension]. Zhonghua xin xue guan bing za zhi. 2011 Jul;39(7):579–615. Chinese. PMID: 22088239. 22088239

[17] Iglesias P, Bajo MA, Selgas R et al. Thyroid dysfunction and kidney disease: An update. Rev Endocr Metab Disord. 2017 Mar;18(1):131–144. 10.1007/s11154-016-9395-7. PMID: 27864708. 10.1007/s11154-016-9395-7 27864708

[18] Kobori H, Ichihara A, Miyashita Y, et al. Mechanism of hyperthyroidism-induced renal hypertrophy in rats. J Endocrinol. 1998 Oct;159(1):9–14. 10.1677/joe.0.1590009. PMID: 9795336; PMCID: PMC2574502. 10.1677/joe.0.1590009 PMC2574502 9795336

[19] Mariani LH, Berns JS. The renal manifestations of thyroid disease. J Am Soc Nephrol. 2012 Jan;23(1):22 – 6. 10.1681/ASN.2010070766. Epub 2011 Oct 21. PMID: 22021708. 10.1681/ASN.2010070766 22021708

[20] Miki H, Oshimo K, Inoue H et al. Thyroid carcinoma in patients with secondary hyperparathyroidism. J Surg Oncol. 1992 Mar;49(3):168 – 71. 10.1002/jso.2930490308. PMID: 1548891. 10.1002/jso.2930490308 1548891

[21] Gauthier BR, Sola-García A, Cáliz-Molina M, et al. Thyroid hormones in diabetes, cancer, and aging. Aging Cell. 2020 Nov;19(11):e13260. 10.1111/acel.13260. Epub 2020 Oct 13. PMID: 33048427; PMCID: PMC7681062. 10.1111/acel.13260 PMC7681062 33048427

[22] Benedetti V, Lavecchia AM, Locatelli M et al. Alteration of thyroid hormone signaling triggers the diabetes-induced pathological growth, remodeling, and dedifferentiation of podocytes. JCI Insight. 2019 Sep 19;4(18):e130249. 10.1172/jci.insight.130249. PMID: 31534055; PMCID: PMC6795387. 10.1172/jci.insight.130249 PMC6795387 31534055

[23] Zou J, Tian F, Zhang Y et al. Association between Thyroid Hormone Levels and Diabetic Kidney Disease in Euthyroid Patients with Type 2 Diabetes. Sci Rep. 2018 Mar16;8(1):4728. 10.1038/s41598-018-22904-7. PMID: 29549262; PMCID: PMC5856822. 10.1038/s41598-018-22904-7 PMC5856822 29549262

[24] Wang J, Li H, Tan M et al. Association between thyroid function and diabetic nephropathy in euthyroid subjects with type 2 diabetes mellitus: a cross-sectional study in China. Oncotarget. 2019 Jan 4;10(2):88–97. 10.18632/oncotarget.26265. PMID: 30719205; PMCID: PMC6349442. 10.18632/oncotarget.26265 PMC6349442 30719205

[25] Qi Q, Zhang QM, Li CJ et al. Association of Thyroid-Stimulating Hormone Levels with Microvascular Complications in Type 2 Diabetes Patients. Med Sci Monit. 2017 Jun4;23:2715–2720. 10.12659/msm.902006. PMID: 28578377; PMCID: PMC5467710. 10.12659/MSM.902006 PMC5467710 28578377

[26] Fei X, Xing M, Wo M, et al. Thyroid stimulating hormone and free triiodothyronine are valuable predictors for diabetic nephropathy in patient with type 2 diabetes mellitus. Ann Transl Med. 2018 Aug;6(15):305. 10.21037/atm.2018.07.07. PMID: 30211193; PMCID: PMC6123216. 10.21037/atm.2018.07.07 PMC6123216 30211193

[27] De Groot LJ. Dangerous dogmas in medicine: the nonthyroidal illness syndrome. J Clin Endocrinol Metab. 1999 Jan;84(1):151 – 64. 10.1210/jcem.84.1.5364. PMID: 9920076. 10.1210/jcem.84.1.5364 9920076

[28] Wiederkehr MR, Kalogiros J, Krapf R. Correction of metabolic acidosis improves thyroid and growth hormone axes in haemodialysis patients. Nephrol Dial Transplant. 2004 May;19(5):1190–7. 10.1093/ndt/gfh096. Epub 2004 Feb 19. PMID: 14993483. 10.1093/ndt/gfh096 14993483

[29] Zoccali C, Tripepi G, Cutrupi S, et al. Low triiodothyronine: a new facet of inflammation in end-stage renal disease. J Am Soc Nephrol. 2005 Sep;16(9):2789–95. 10.1681/ASN.2005040356. Epub 2005 Jul 20. PMID: 16033857. 10.1681/ASN.2005040356 16033857

